# P381: Legionella priority control: development of a comprehensive tool for healthcare settings

**DOI:** 10.1186/2047-2994-2-S1-P381

**Published:** 2013-06-20

**Authors:** P Del Giudice, L Arnoldo, D Goi, R Cocconi, B Casini, G Gurnari, U Moscato, ML Cristina, S Brusaferro

**Affiliations:** 1 University of Udine, Italy; 2 Azienda Ospedaliero-Universitaria Santa Maria della Misericordia, Udine, Italy; 3 University of Pisa, Pisa, Italy; 4 Catholic University of the Sacred Heart, Rome, Italy; 5 University of Genoa, Genoa, Italy

## Introduction

Despite scientific and technological advances, Legionella continues to be a major threat to patients in healthcare settings. To be effective the control of Legionella need a competent attention based on a correct risk assessment and on consequent operative choices.

## Objectives

A tool called Legionella Priority Control [LPC], was designed to be comprehensive, easily implementable and able to define intervention priorities in the healthcare setting.

## Methods

LPC was developed within a project funded by the Italian Ministry of University through the following stages:

- Review of literature to identify all the possible critical points in Legionella control;

- Classification of all selected critical points in three independent areas of interest: management, water distribution system and patient susceptibility;

- Identification of several critical points for each area;

- Split of each critical point into items to be answered in form of questionnaire;

- Classification of items through a multidisciplinary consensus into 4 risk categories (1 - best practice/low to no risk, 2 - possible Legionella presence (not evidence based)/medium risk, 3 - probable Legionella presence (evidence based)/high risk, 4 - demonstrated Legionella presence/very high risk);

- Analysis of the 3 areas scores in order to define a general risk score and a structure related risk score;

- Use of a risk matrix (including a feasibility score to be assigned by local practitioners linked to the setting) to define final priorities.

## Results

LPC includes:

- A check list of items which, once filled in, allows the definition of existing problems (3 areas of interest, 15 critical points and 76 items);

- A risk matrix which allows definition of an initial list of priorities;

- A subjective feasibility score which allows definition of priorities tailored to the local setting.

## Conclusion

LPC offers to the infection control practitioner an easier yet comprehensive way to define priorities for Legionella control tailored to the local healthcare setting.

## Disclosure of interest

None declared

